# Supplementary material for: Microstructural Considerations of a Multi-Pass Rolled Ti-Nb-Ta-Zr Alloy
Source: Materials (Basel). 2023 Apr 19;16(8):3208. doi: 10.3390/ma16083208 (PMC10144209; doi:10.3390/ma16083208)
Supplement: Supplementary file 1 [file materials-16-03208-s001.zip › materials-2311041-supplementary.pdf]

Supplementary Material

Figures S1 – S12 show the detailed zooms of cumulative diffraction peaks and the Rietveld plots for the Ti29Nb-9Ta-10Zr alloy corresponding to all studied stages.

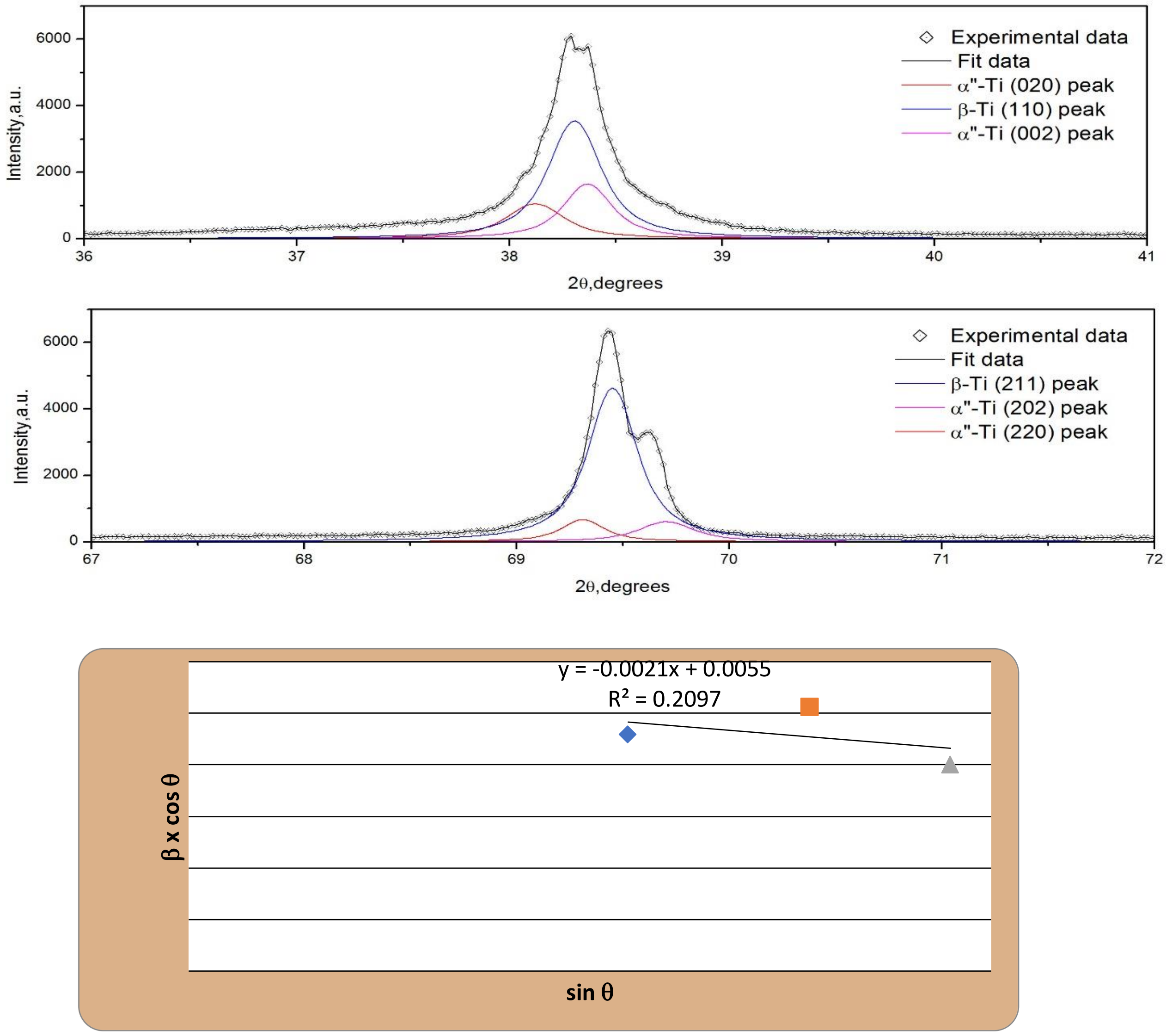

Figure S1 - The detailed zooms of cumulative diffraction peaks (top) and the Rietveld plot (down) for the Ti29Nb-9Ta-10Zr alloy corresponding to initial state.

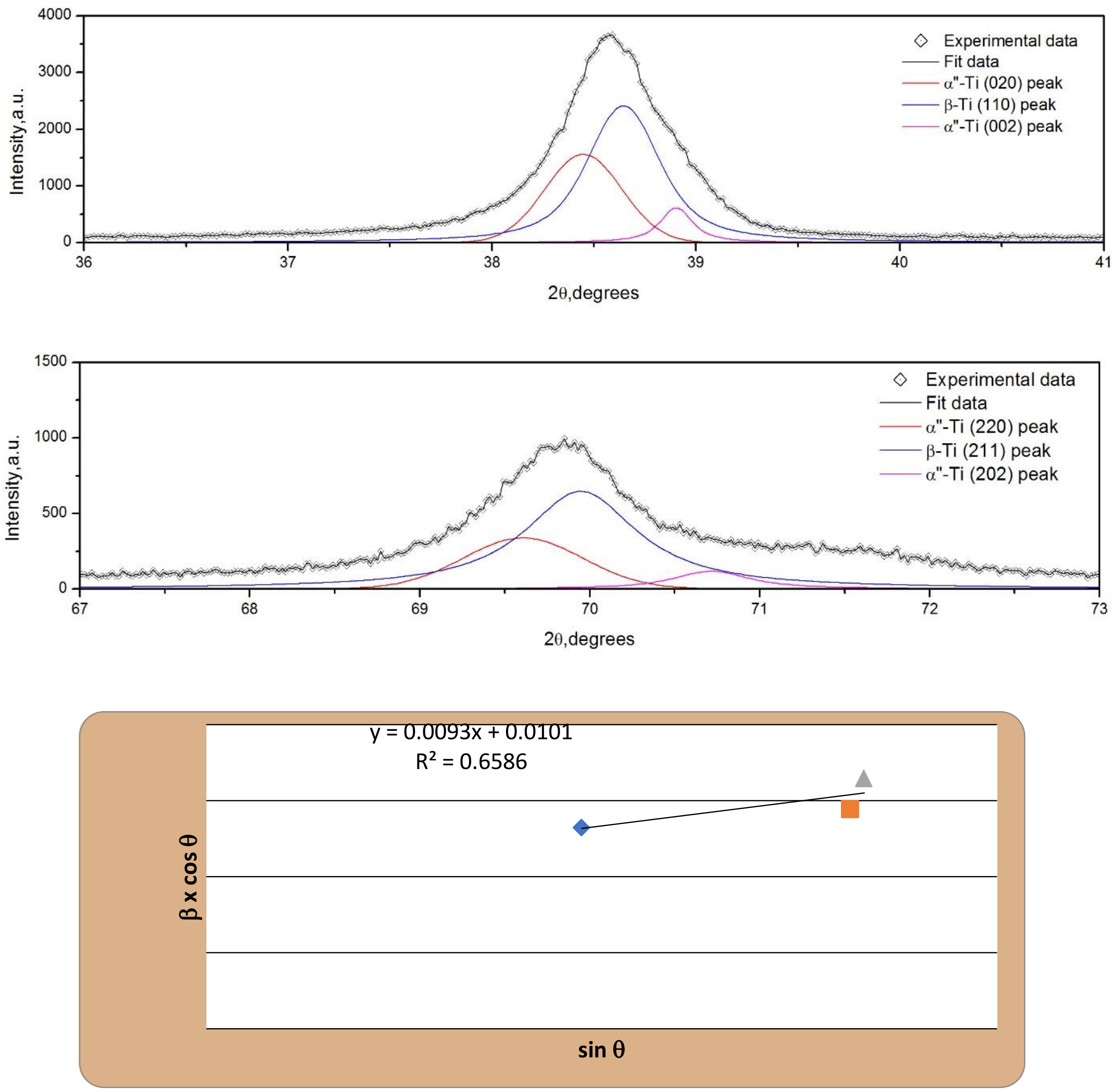

Figure S2 - The detailed zooms of cumulative diffraction peaks (top) and the Rietveld plot (down) for the Ti29Nb-9Ta-10Zr alloy corresponding to MPR (20%) state.

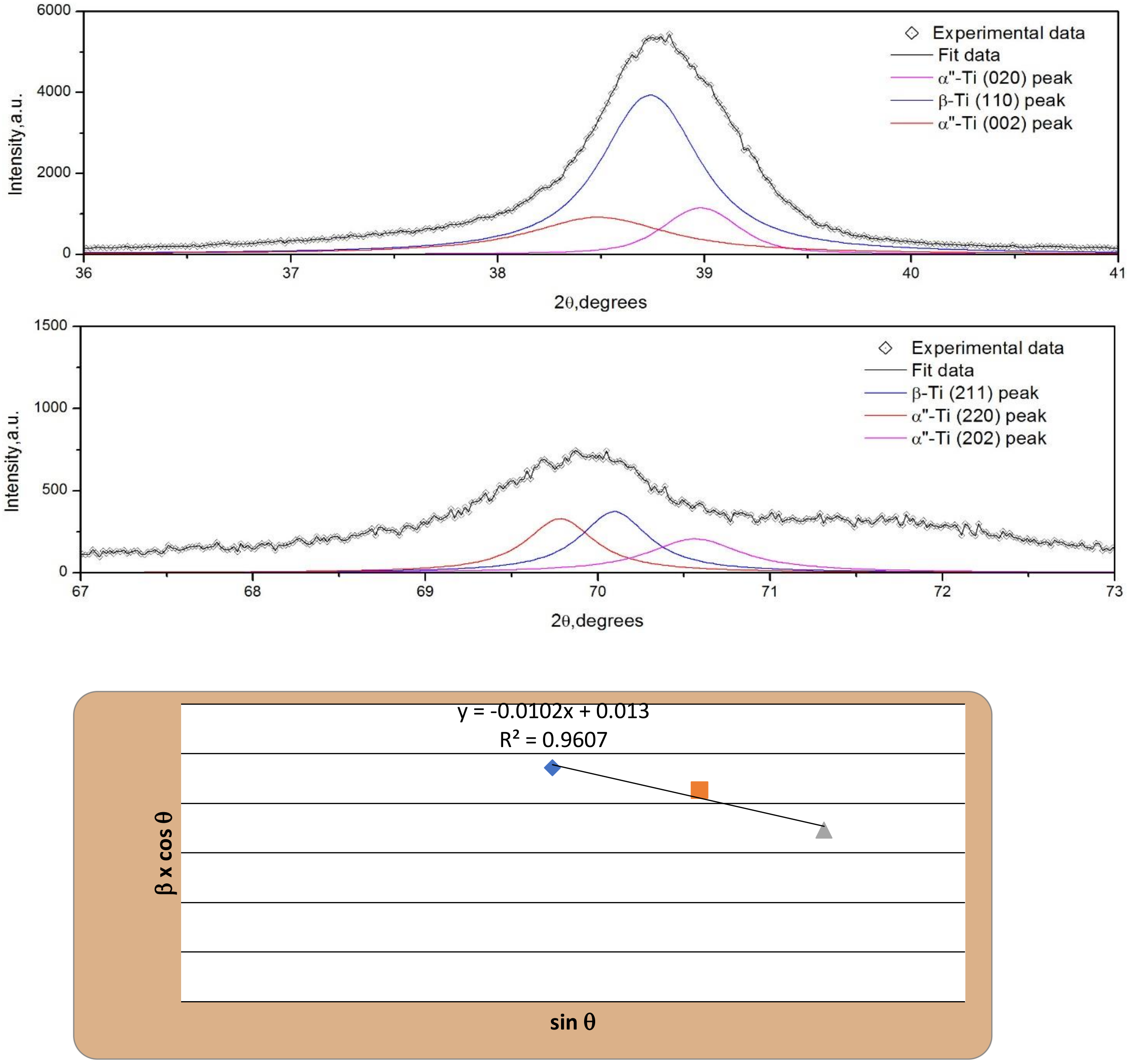

Figure S3 - The detailed zooms of cumulative diffraction peaks (top) and the Rietveld plot (down) for the Ti29Nb-9Ta-10Zr alloy corresponding to MPR (40%) state.

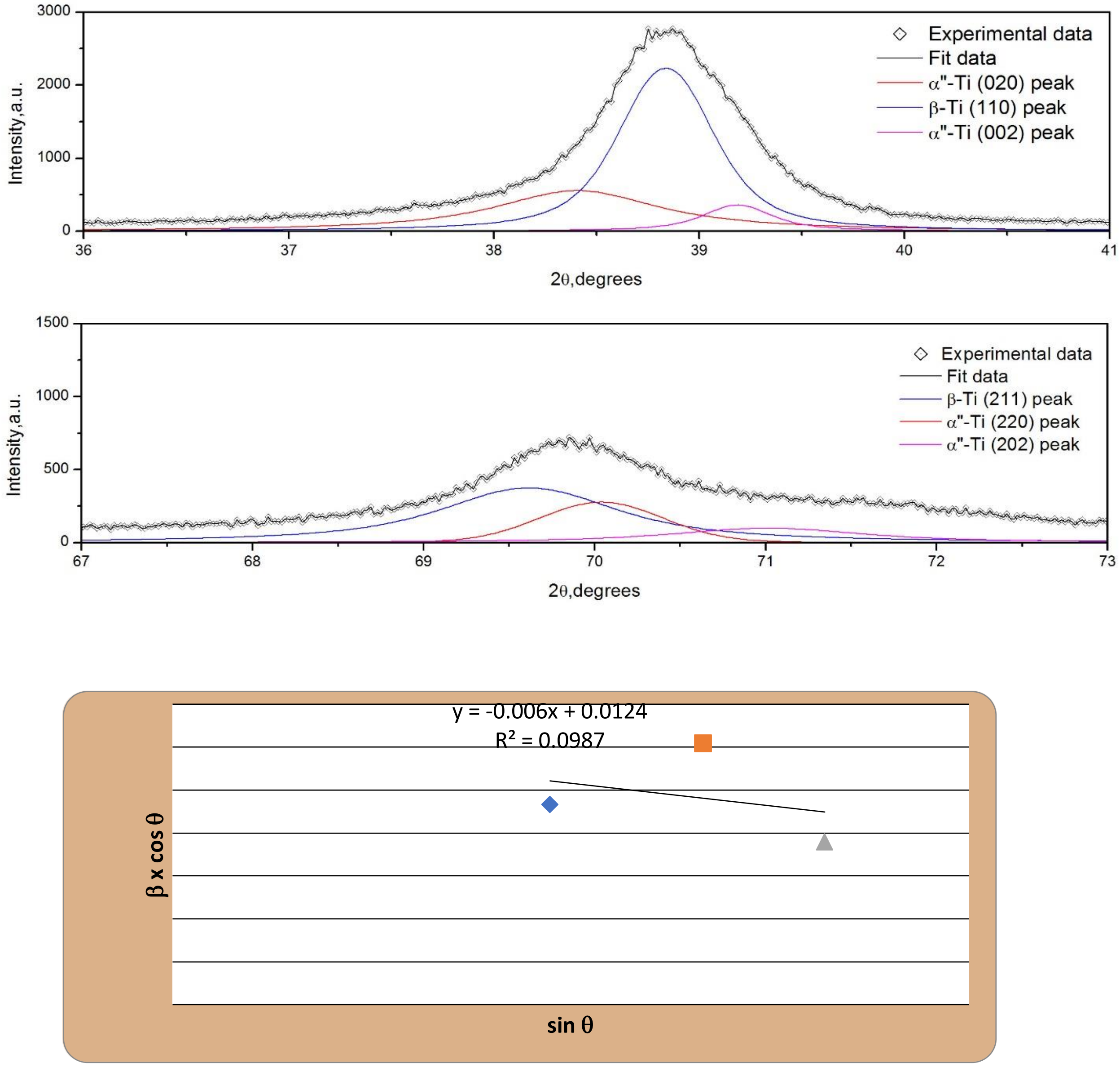

Figure S4 - The detailed zooms of cumulative diffraction peaks (top) and the Rietveld plot (down) for the Ti29Nb-9Ta-10Zr alloy corresponding to MPR (60%) state.

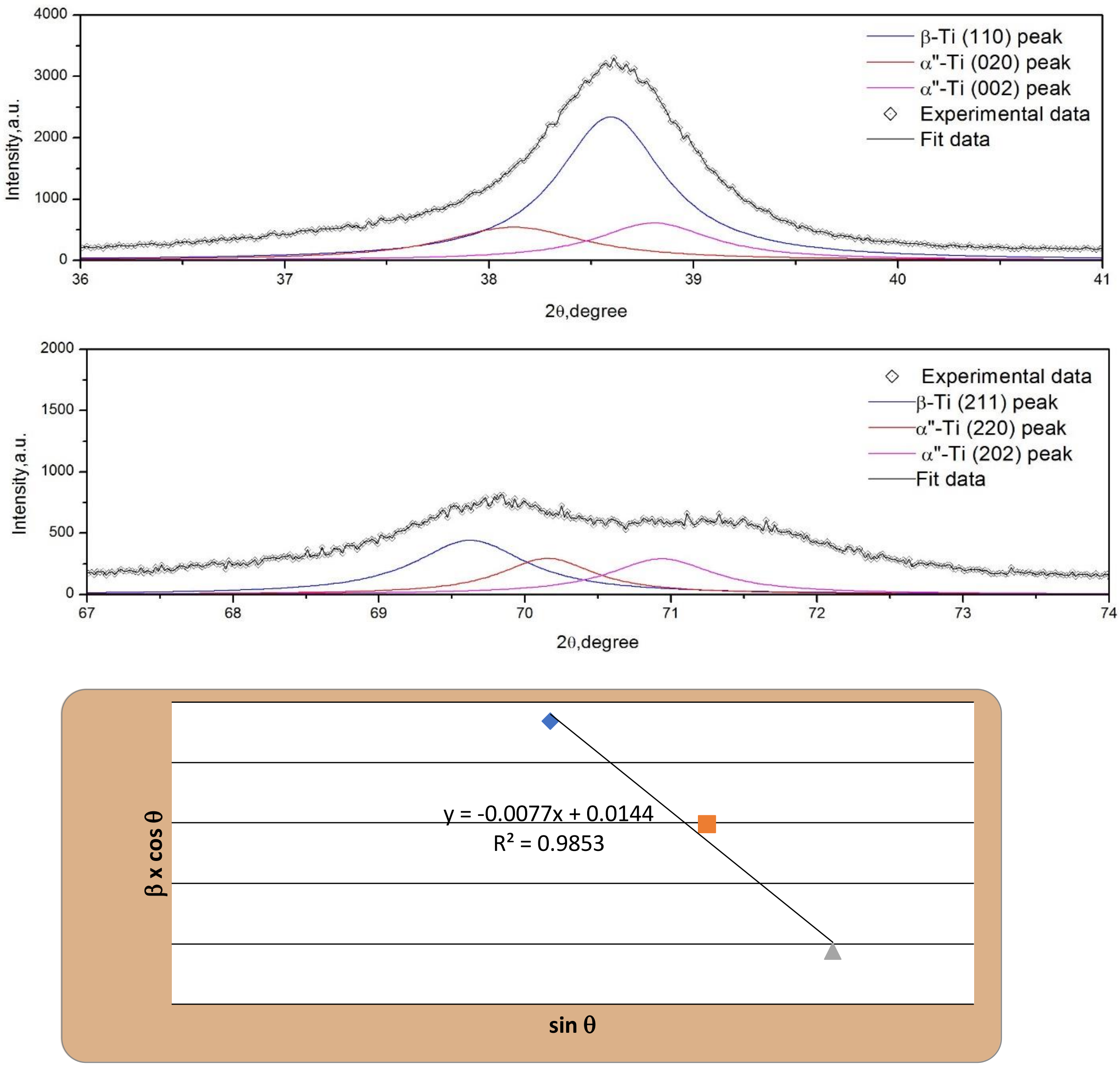

Figure S5 - The detailed zooms of cumulative diffraction peaks (top) and the Rietveld plot (down) for the Ti29Nb-9Ta-10Zr alloy corresponding to MPR (80%) state.

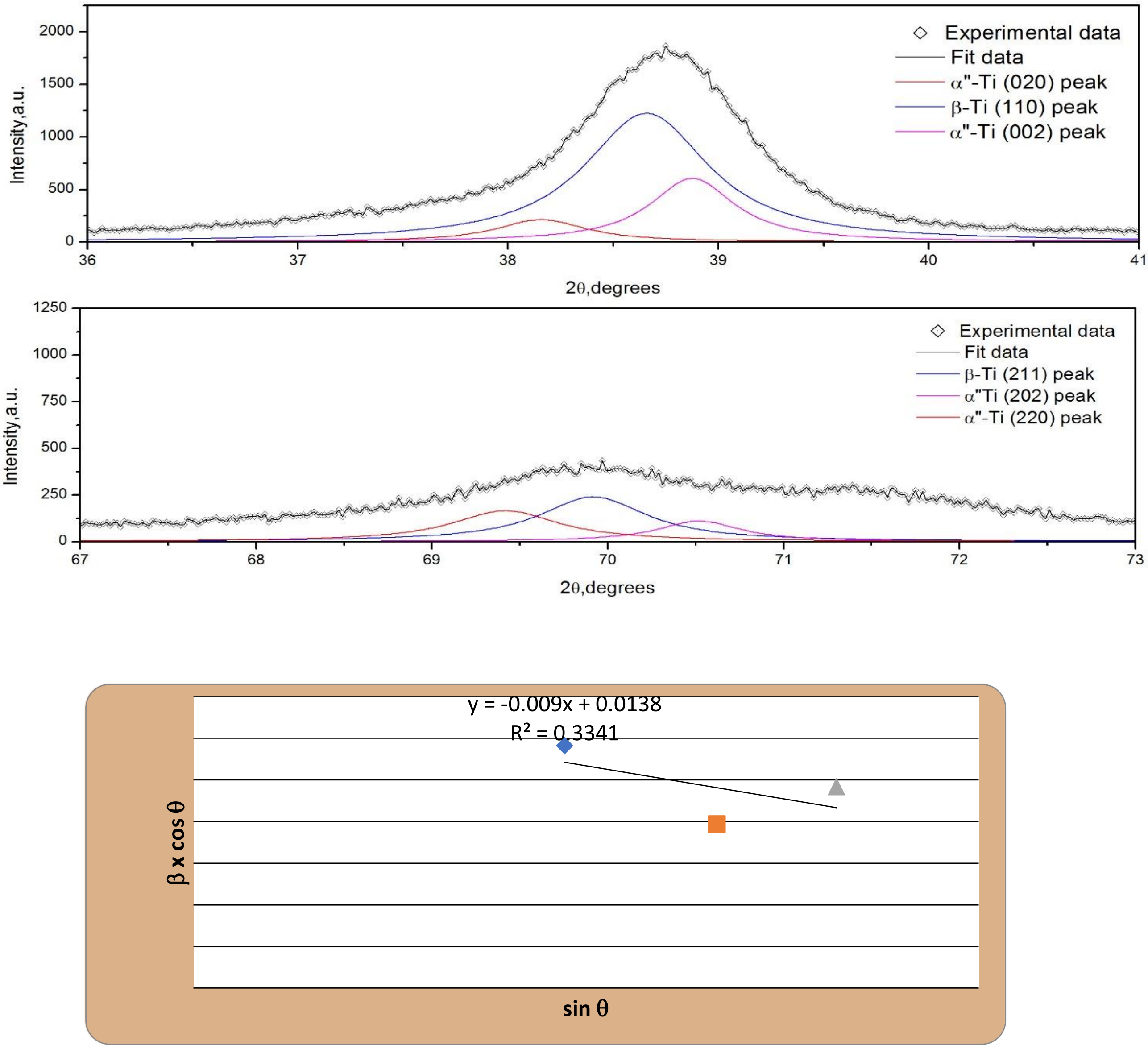

Figure S6 - The detailed zooms of cumulative diffraction peaks (top) and the Rietveld plot (down) for the Ti29Nb-9Ta-10Zr alloy corresponding to MPR (90%) state.

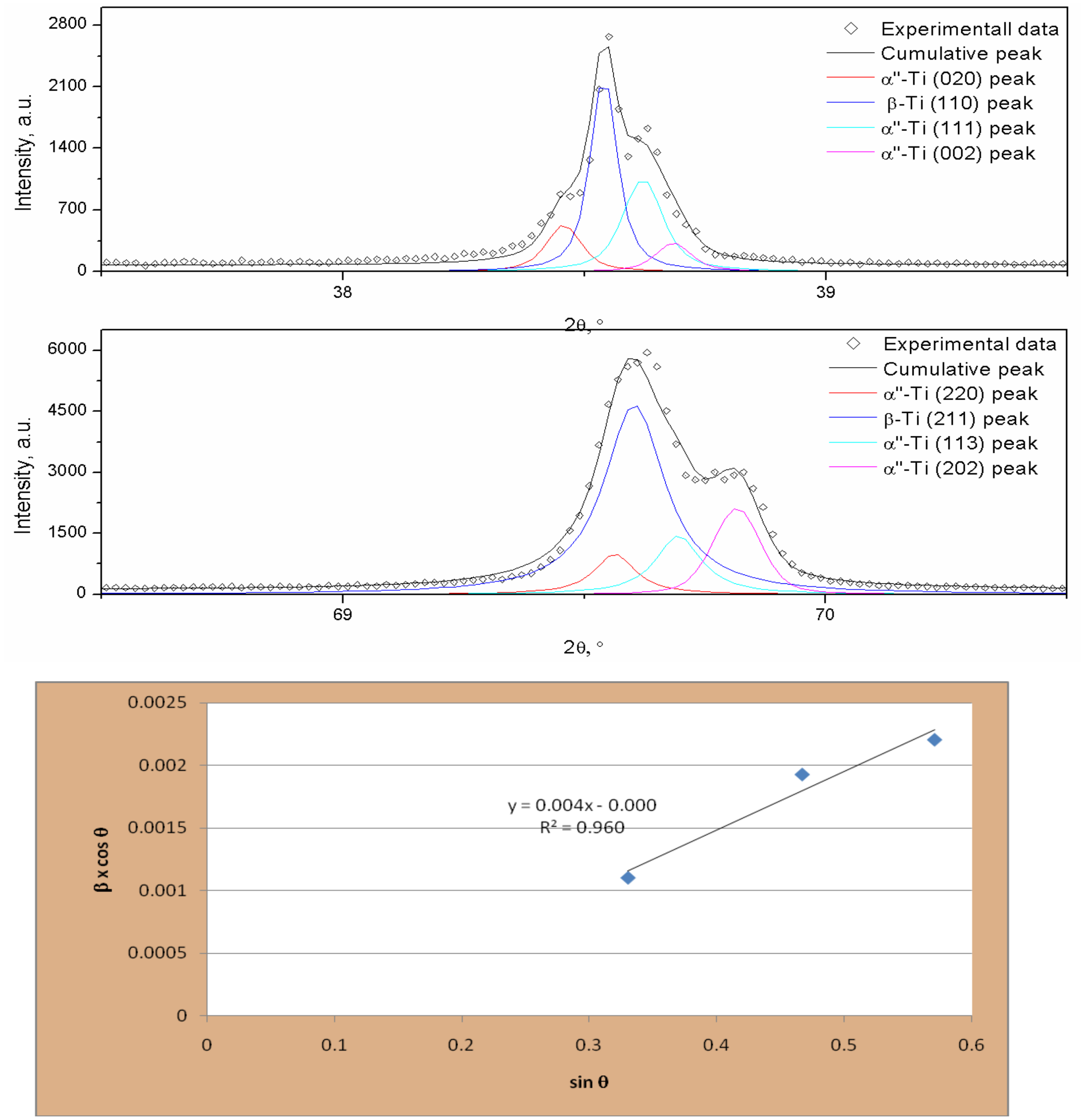

Figure S7 - The detailed zooms of cumulative diffraction peaks (top) and the Rietveld plot (down) for the Ti29Nb-9Ta-10Zr alloy corresponding to MPR (90%) + R1(780°C/5 min/w.q.) state.

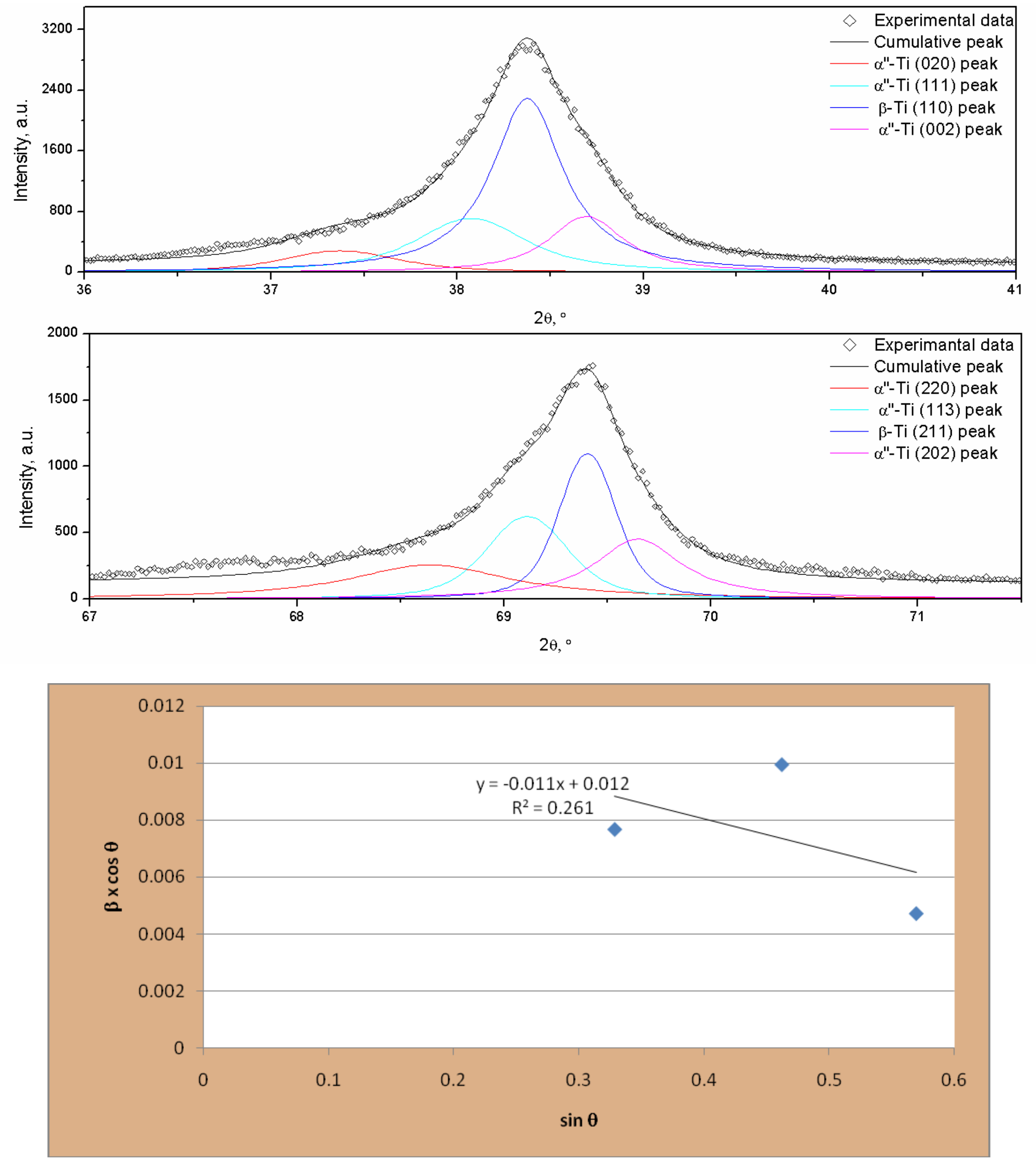

Figure S8 - The detailed zooms of cumulative diffraction peaks (top) and the Rietveld plot (down) for the Ti29Nb-9Ta-10Zr alloy corresponding to MPR (90%) + R2 (830°C/5 min/w.q.) state.

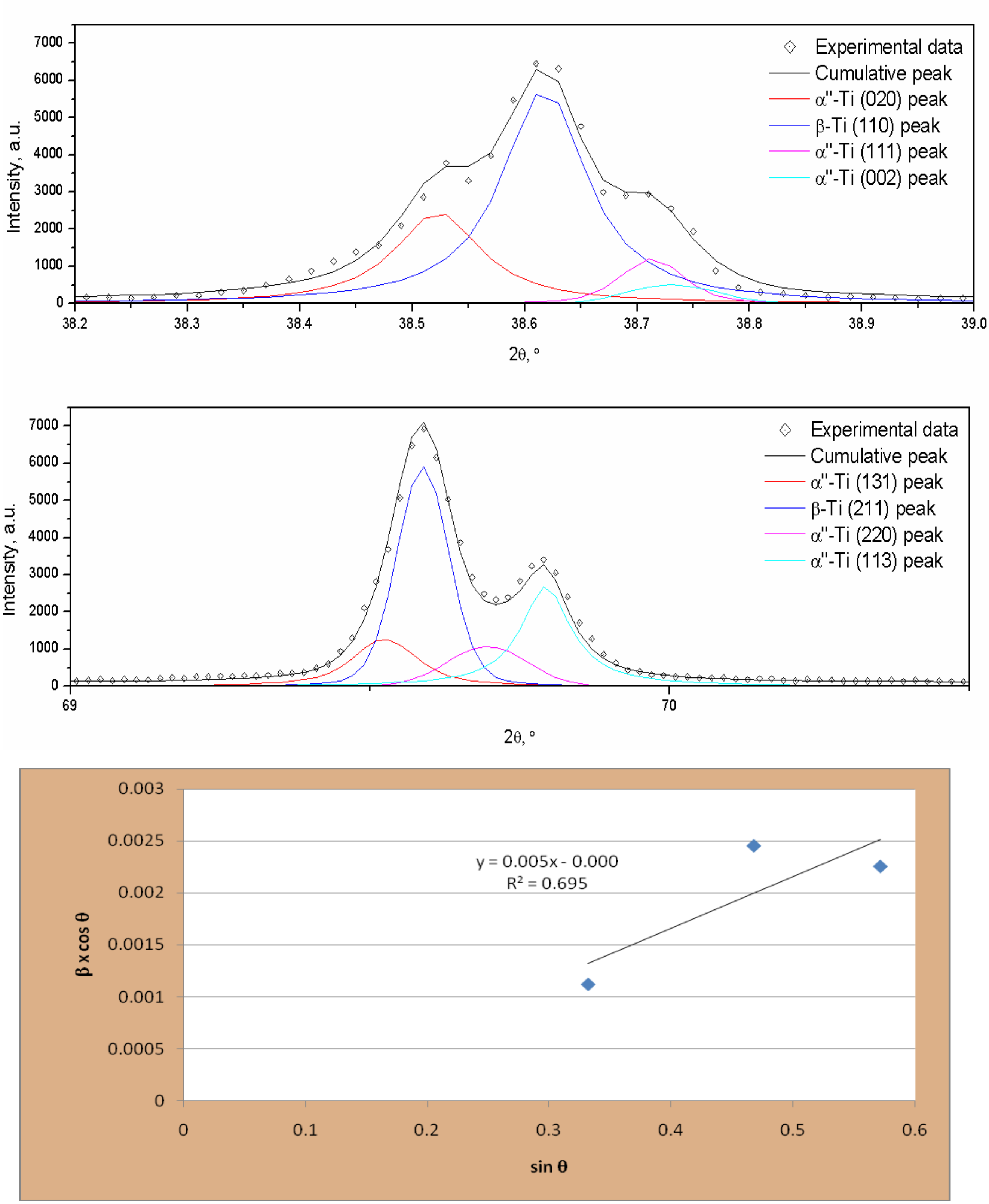

Figure S9 - The detailed zooms of cumulative diffraction peaks (top) and the Rietveld plot (down) for the Ti29Nb-9Ta-10Zr alloy corresponding to MPR (90%) + R3 (880°C/5 min/w.q.) state.

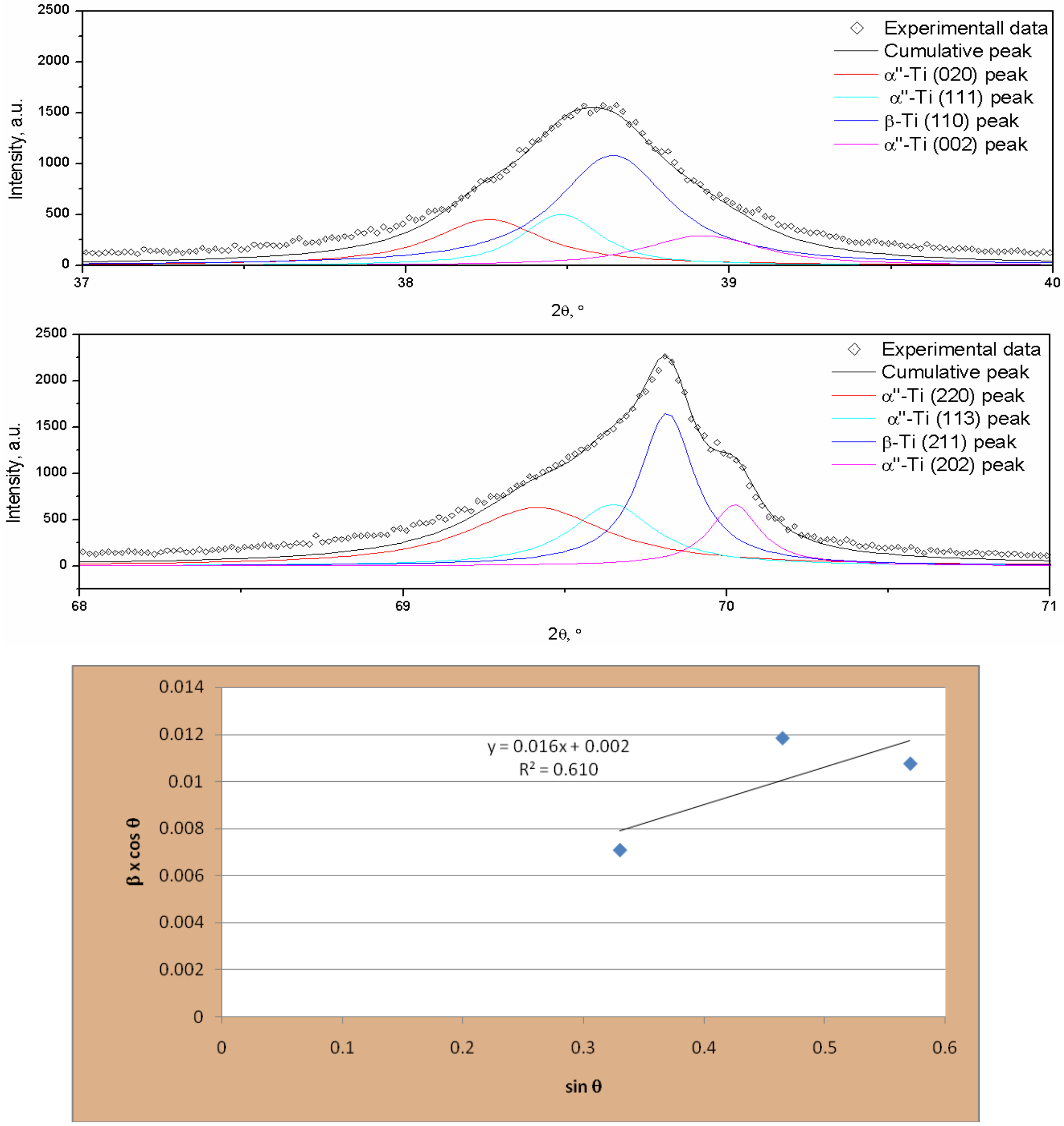

Figure S10 - The detailed zooms of cumulative diffraction peaks (top) and the Rietveld plot (down) for the Ti29Nb-9Ta-10Zr alloy corresponding to MPR (90%) + R1 (780°C/5 min/w.q.) + A (400°C/5 min/w.q.) state.

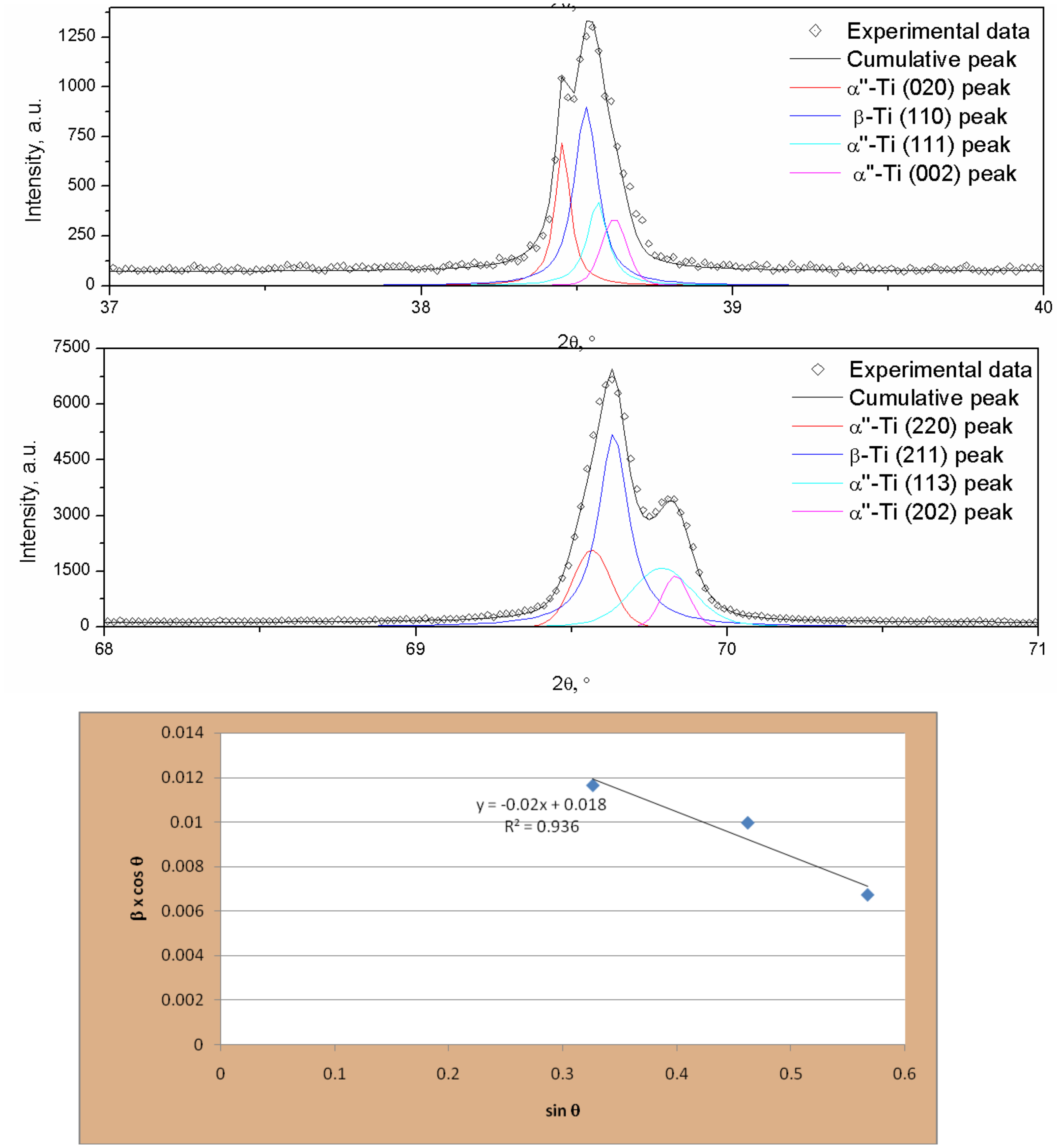

Figure S11 - The detailed zooms of cumulative diffraction peaks (top) and the Rietveld plot (down) for the Ti29Nb-9Ta-10Zr alloy corresponding to MPR (90%) + R2 (830°C/5 min/w.q.) + A (400°C/5 min/w.q.) state.

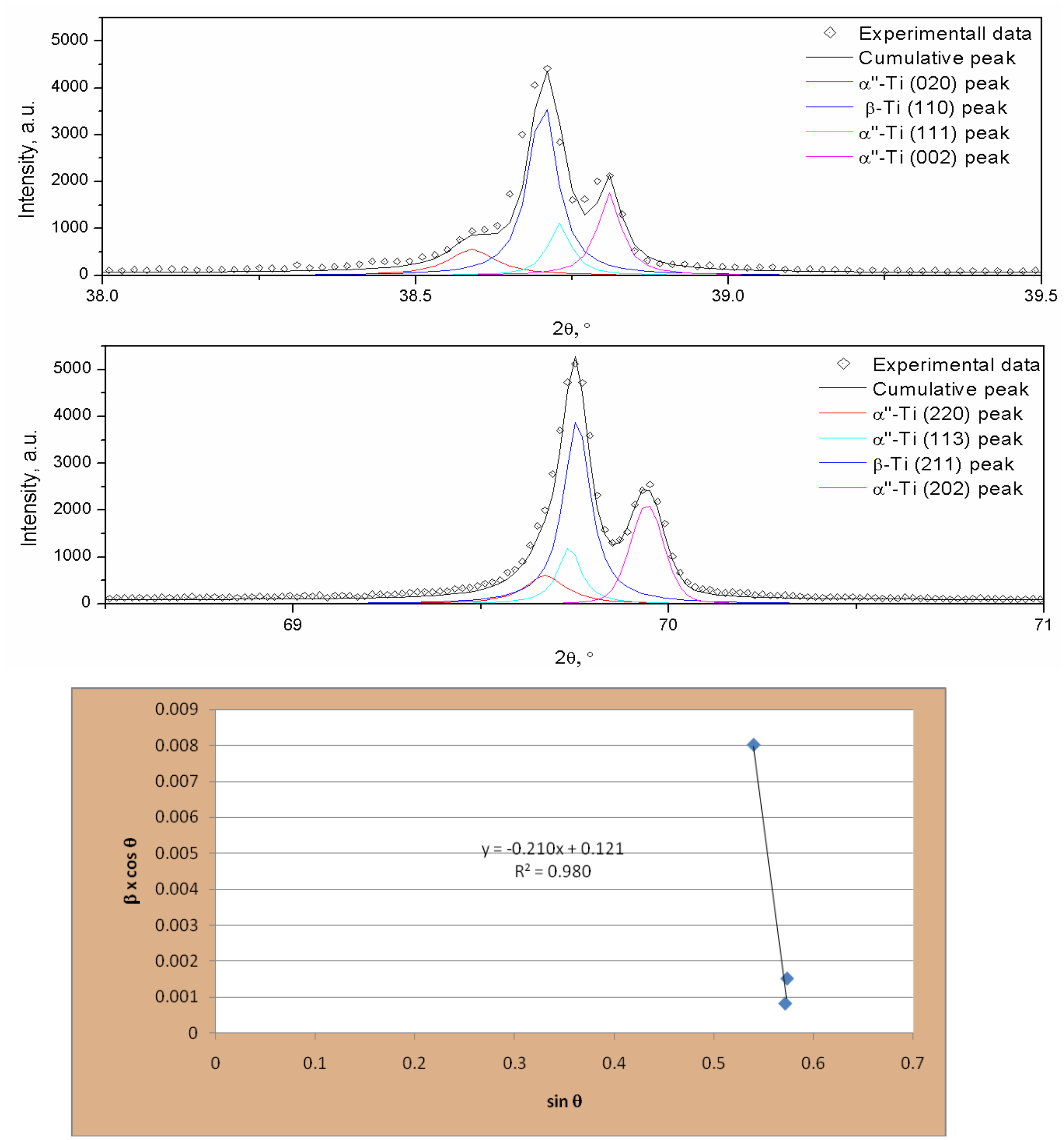

Figure S12 - The detailed zooms of cumulative diffraction peaks (top) and the Rietveld plot (down) for the Ti29Nb-9Ta-10Zr alloy corresponding to MPR (90%) + R3 (880°C/5 min/w.q.) + A (400°C/5 min/w.q.) state.
